# Isolated cerebral aspergillus abscess as a complication of pulmonary alveolar proteinosis in a child

**DOI:** 10.1186/s41016-019-0165-8

**Published:** 2019-07-18

**Authors:** Rachna Khera, Venkateswara Rao, Mohan Krishna Pasam, Ravindranath Tagore, Sudha S. Murthy, Challa Sundaram

**Affiliations:** 1 Department of Laboratory Medicine, Basavatarakam Indo-American Cancer Hospital and Research Institute, Hyderabad, Telangana 500034 India; 2 Department of Neurosurgery, Basavatarakam Indoamerican Cancer Hospital and Research Institute, Hyderabad, Telangana 500034 India

**Keywords:** Pulmonary alveolar proteinosis, Opportunistic infection, *Aspergillus fumigatus*, Cerebral abscess

## Abstract

**Abstract:**

**Background:**

Pulmonary alveolar proteinosis (PAP) poses a risk of opportunistic infections with a variety of organisms with *Nocardia* being the most common pathogen followed by mycobacteria and fungi.

**Case presentation:**

A 7-year-old female child, presented with headache and multiple episodes of vomiting. There was no fever or altered sensorium. On examination, there were no focal deficits or cranial nerve palsies. An MRI brain showed a small T2 hyperintense lesion in the left superior parietal lobe suggestive of an abscess. She was diagnosed as PAP based on CT chest and bronchioloalveolar lavage 7 months earlier and treated with corticosteroids. A left parieto-occipital craniotomy was done with drainage of abscess and abscess wall excision. Histopathology revealed a suppurative lesion with slender septate acute angle branching hyphae which were positive on fungal stains. Culture done on the pus was positive for *Aspergillus fumigatus*. The patient was treated with voriconazole and stable at 1 year follow-up.

**Conclusion:**

Opportunistic infections are common in patients diagnosed with PAP. High index of clinical suspicion and early diagnosis are important for favorable outcome.

## Background

Pulmonary alveolar proteinosis (PAP) is a rare disease characterized by accumulation of lipo-proteinaceous surfactant material in the alveolar and bronchiolar spaces because of defective clearance by alveolar macrophages [1–3]. It is known to occur in three distinct clinical forms: primary/congenital, secondary, and autoimmune. Autoimmune PAP is the most common form of this disease (90%) and is the result of the formation of anti-granulocyte-macrophage colony stimulating factor (GM-CSF) antibodies [2–5]. Primary/congenital PAP is associated with genetic mutations in surfactant proteins or GM-CSF mutations and usually occurs in children. Secondary PAP is associated with functional impairment of alveolar macrophages that is seen in relation to hematological malignancies, exposure to infections and toxins, allogenic stem cell transplantation, and others [2, 3, 6].

About 5% of patients having PAP present with opportunistic infections^,^ most commonly with, *Nocardia, Mycobacteria* followed by fungal infections [2, 5, 7, 8]. A significant number of disseminated infections involve the central nervous system (CNS) with infective organism being fungus [9]. The fungal infections in association with PAP are most commonly due to *Cryptococcus*, followed by *Aspergillus*, *Histoplasma*, and others [2, 9]. Fungal infections associated with PAP have high mortality and usually diagnosed at autopsy [2, 9]. We report a child with autoimmune PAP and isolated cerebral *Aspergillus* abscess with a favorable outcome due to its extreme rarity.

## Case presentation

**Fig. 1:**
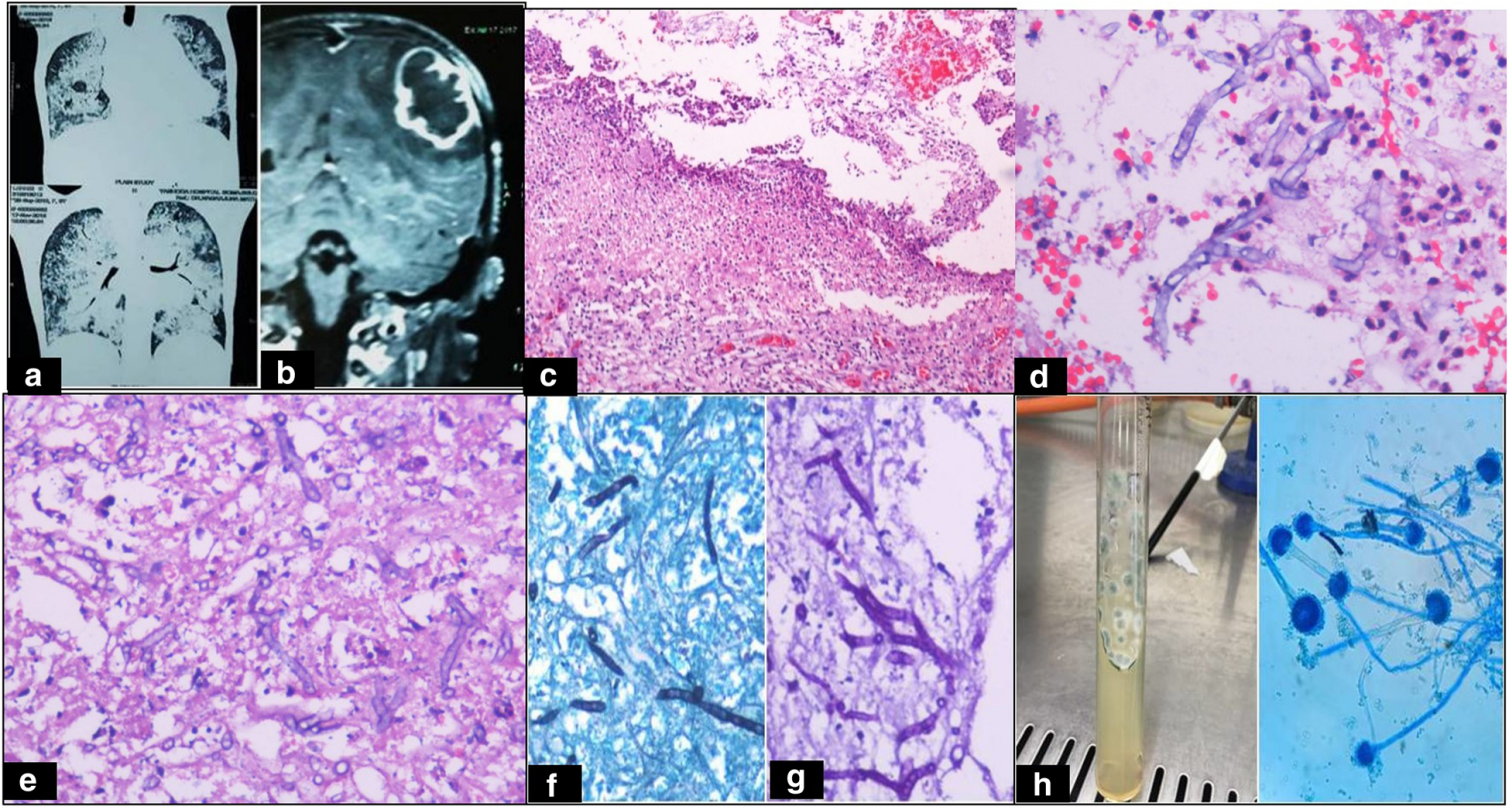
**a** Plain CT chest showing bilateral diffuse miliary ground glass opacities. **b** MRI brain showing T2 hyperintense ring-enhancing lesion in the left superior parietal lobe suggestive of an abscess. **c** H&E × 40 Abscess wall with dense acute inflammatory exudates. **d** H&E × 400 showing multiple filamentous septate hyphae with acute angle branching. **e** H&E × 400 numerous fungal hyphae embedded in the exudates. **f** Silver methenamine stain × 400 demonstrating grey black colored fungal organisms. **g** PAS stain × 400 showing magenta colored organisms. **h** Sabourad dextrose agar demonstrating greenish blue colonies with white edges classical for *A. fumigatus*. Lactophenol cotton blue stained smear demonstrating fruiting bodies

A 7-year-old female child presented with headache, multiple episodes of vomiting, and speech disturbances. She had no fever, altered sensorium, or focal neurological deficits. She was conscious and coherent with normal motor and sensory functions. She was diagnosed to have PAP on CT chest and bronchiole alveolar lavage (BAL) in another hospital 7 months earlier and was on treatment with corticosteroids (Fig. 1a). There was neutrophil leukocytosis (6900/cu mm). Rest of the hematological and biochemical investigations were within normal limits. MRI brain showed a T2 hyperintense lesion in the left superior parietal lobe (Fig. 1b). A diagnosis of tuberculoma was made and she was treated with anti-tuberculous treatment. However, she did not respond even after three weeks and was symptomatic. Left parieto-occipital craniotomy was done and per-operative smears showed necrotic material with neutrophils and septate hyphae. Abscess drainage and abscess wall excision were done. Histopathology revealed a suppurative lesion with neutrophils and mononuclear cells (Fig. 1c). Amidst the necrotic material were seen delicate septate hyphae with acute angle branching (Fig. 1d–f). The hyphae were highlighted by Gomori methenamine silver (GMS) and periodic acid Schiff (PAS) stains (Fig. 1g). *Aspergillus fumigatus* was isolated on culture from the pus. There were grey to blue green colonies and microscopy showed conidia (fruiting bodies) (Fig. 1h). Culture of sputum and bronchoalveolar lavage was negative. Further, the patient was treated with voriconazole with uneventful follow-up till date (1 year).

## Discussion

Complications of PAP include opportunistic infections, interstitial fibrosis, and rare instances of emphysematous bullae and pneumothorax [10]. Infections associated with PAP may precede, follow or may also present in concurrence. PAP was reported as the initial presentation in 33% patients as seen in our patient [9]. Majority of the infections were reported in adults with a mean age of 39 years and male predominance [9]. Our patient was a 7-year-old female child.

First extensive review of infections in patients with PAP was done by Seymour et al. who reported that majority were by *Nocardia* followed by *Mycobacteria*, *Cryptococcus, Histoplasma*, *Aspergillus*, and others [2]. Punatar et al. in 2012, reviewed a total of 75 cases from 1950 to July 2010 and identified *Nocardia* (43%) as the most common pathogen followed by mycobacteria (37%) and fungi (20%) [9]. Of the 15 cases of fungal infections in their study, 5 were due to *Cryptococcus* and 4 each due to *Aspergillus* and *Histoplasma*. The most common site of infection was lung followed by the brain [9]. CNS involvement was seen usually in disseminated cases [9]. Our patient had cerebral *Aspergillus* abscess as the sole manifestation 7 months after the diagnosis of PAP.

Cryptococcosis is the most common infection reported in association with PAP; however, the other fungal infections include those due to *Aspergillus*, *Histoplasma*, *Mucor*, *Blastomyces Coccidioides*, and *Pneumocystis* [1, 2, 9, 11, 12]. They are all mycoses controlled by macrophages upon inhalation [12]. Susceptibility to infections is found to be multi-factorial. Possible causes include impaired macrophage function and impaired host defense [12]. When autoantibodies block the GM-CSF pathway, differentiation and function of alveolar macrophages are impaired and the ability to clear surfactants is diminished [2, 11, 12]. As a result, pulmonary alveoli accumulate periodic acid-Schiff (PAS)-positive proteinaceous surfactant components which act as a medium for infection [2, 11, 12]. Since autoantibodies to GM-CSF cause defects in chemotaxis, adhesion, phagocytosis, microbicidal activity, and phagolysosome fusion of alveolar macrophages, patients with PAP are at risk for infections from a variety of respiratory microorganisms including fungal species [2, 11, 12]. In our patient, screening for mutations of surfactant protein or assay for GM-CSF autoantibodies was not done. Corticosteroids were administered after the diagnosis of PAP and possibly it would have augmented the risk for opportunistic infection with *Aspergillus.*

The first case of CNS aspergillosis in a patient with PAP was described by Björkholm et al. [13]. Kourkoumpetis et al. reported 14 cases of CNS aspergillosis and reviewed 123 reported cases from literature [14]. In their report, CNS aspergillosis was associated with a wide variety of co-morbidities including diabetes mellitus, human immunodeficiency virus infection, hematological malignancies, transplant, etc., but none with PAP. Neutropenia was found to be a major contributor to the pathogenesis [14]. However, in the present case, the patient did not have neutropenia. Qualitative rather than quantitative defect in neutrophilic function may be attributed to the opportunistic infections [14]. Diagnosis of CNS aspergillosis is difficult as the clinical symptoms are non-specific [14]. Our patient was initially diagnosed to have CNS tuberculoma on MRI and was treated with ATT before she underwent surgery. *Aspergillus* f*umigatus* was found to be the most common pathogen in CNS Aspergillosis as seen in our patient [14, 15]. Mortality rate was high in patients with CNS aspergillosis (28% in who underwent neurosurgery and 67% in who did not undergo neurosurgery) [14]. However, Dotis et al. reported that there is a significant difference in mortality in cases reported before and after 1990 (82% and 39% respectively) which can be attributed to the introduction of amphotericin B and aspergillus sensitive azoles like voriconazole [15]. Our patient underwent surgical excision and received voriconazole and stable at 1 year follow-up.

## Conclusion

Pulmonary alveolar proteinosis is one of the risk factors for opportunistic infections. High index of clinical suspicion for unusual pathogens like fungus and uncommon sites like CNS should always be considered in patients with PAP. Timely pathological diagnosis and a combination of surgical and antifungal therapy improve the outcome in patients with CNS aspergillosis in association with PAP. This case is reported for its rarity and to emphasize the importance of early diagnosis and treatment for a better clinical outcome.

## Data Availability

Data sharing not applicable to this article as no datasets were generated or analyzed during the current study.

## Abbreviations

BAL: Bronchoalveolar lavage
CNS: Central nervous system
GM-CSF: Granulocyte-macrophage colony-stimulating factor
GMS: Gomori methenamine silver
PAP: Pulmonary alveolar proteinosis
PAS: Periodic acid schiff
